# Skilled Reaching and Grasping in the Rat: Lacking Effect of Corticospinal Lesion

**DOI:** 10.3389/fneur.2014.00103

**Published:** 2014-06-20

**Authors:** Bror Alstermark, Lars-Gunnar Pettersson

**Affiliations:** ^1^Section of Physiology, Department of Integrative Medical Biology, Umeå University, Umeå, Sweden; ^2^Department of Physiology, Institute of Neuroscience and Physiology, Sahlgrenska Academy at University of Gothenburg, Gothenburg, Sweden

**Keywords:** skilled forelimb movements, reaching, grasping, corticospinal tract lesion, reticulospinal, interneuron, motorneuron

## Abstract

The corticospinal system is a major motor pathway in the control of skilled voluntary movements such as reaching and grasping. It has developed considerably phylogenetically to reach a peak in humans. Because rodents possess advanced forelimb movements that can be used for reaching and grasping food, it is commonly considered that the corticospinal tract (CST) is of major importance for this control also in rodents. A close homology to primate reaching and grasping has been described but with obvious limitations as to independent digit movements, which are lacking in rodents. Nevertheless, it was believed that there are, as in the primate, direct cortico-motoneuronal connections. Later, it was shown that there are no such connections. The fastest excitatory pathway is disynaptic, mediated via cortico-reticulospinal neurons and in the spinal cord the excitation is mainly polysynaptically mediated via segmental interneurons. Earlier behavioral studies have aimed at investigating the role of the CST by using pyramidotomy in the brainstem. However, in addition to interrupting the CST, a pyramidal transection abolishes the input to reticulospinal neurons. It is therefore not possible to conclude if the deficits after pyramidotomy result from interruption of the CST or the input to reticulospinal neurons or both. We have re-investigated the role of the CST by examining the effect of a CST lesion in the C1–C2 spinal segments on the success rate of reaching and grasping. This lesion spares the cortico-reticulospinal pathway. In contrast to investigations using pyramidal transections, the present study did not demonstrate marked deficits in reaching and grasping. We propose that the difference in results can be explained by the intact cortical input to reticulospinal neurons in our study and thus implicate an important role of this pathway in the control of reaching and grasping in the rat.

## Introduction

Skilled reaching and grasping are among the most complex voluntary movements that many different species of animals perform for their daily living and survival. The corticospinal tract (CST) plays a major role in the control of skilled voluntary movements such as reaching and grasping in higher species (1–5). This function has developed during phylogeny and has reached its peak in man (3, 6, 7). Rodents also perform skilled forelimb movements and it is generally accepted that the CST has an important role for reaching and digit grasping (8, 9). At first, microcircuit analysis using anatomical tracing and electrophysiology suggested, as in the primate, the existence of direct cortico-motoneuronal connections (10). These results further emphasized the idea that independent digit movements can only be performed if there are direct cortico-motoneuronal connections. Later, it was shown that there are no such connections in rodents (11, 12). It has been shown that the fastest excitatory pathways from motor cortex in the rat are mediated disynaptically via a cortico-reticulospinal–motoneuronal pathway (11). In the earlier behavioral studies, the lesion was made in the pyramid (8) at the brainstem level or in the motor cortex (9) and therefore interrupted not only the CST, but also cortico-reticular projections. We have now investigated the contribution of the CST in the rat by comparison of the success rate of reaching and grasping of a small morsel of food with the forepaw before and after transection of the axons in the dorsal column in the C1/C2 segmental border. This lesion eliminates the corticospinal input to the segmental interneurons (sINs), but spares the input to reticulospinal neurons as shown in Figure 3D (11).

## Materials and Methods

### Ethics

The study was conducted in accordance with national laws (The Swedish Animal Protection Act and Animal Protection Ordinance) including approval by a regional ethical committee (Swedish Board of Agriculture).

### Subjects and housing

**Figure 1:**
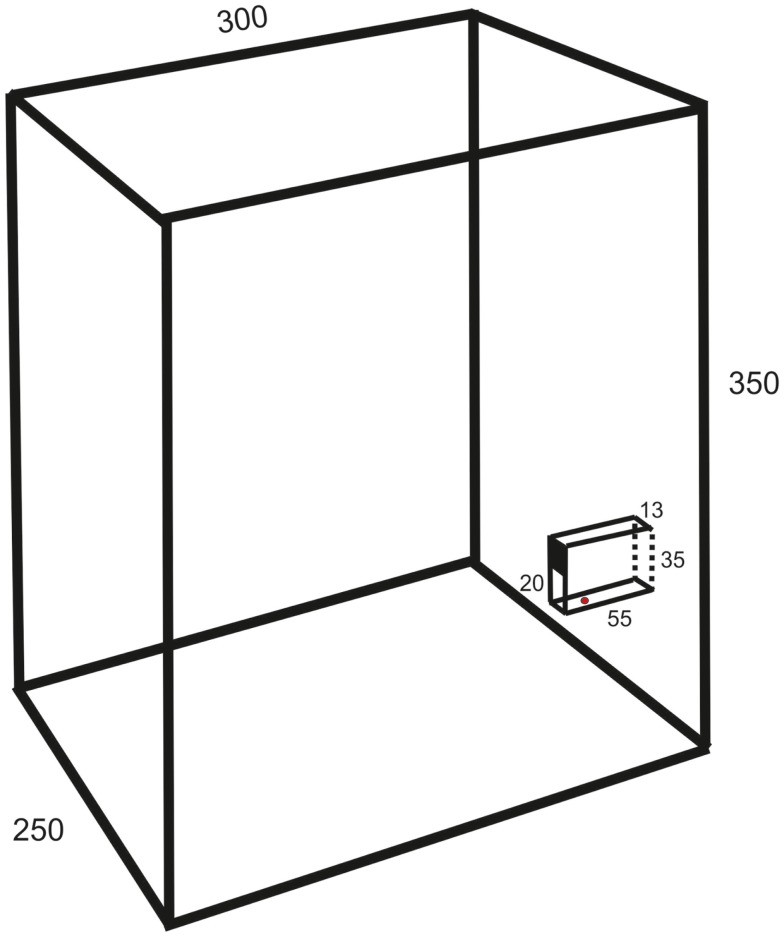
**Schematic drawing of the test box (oblique lateral view; measurements in millimeters) showing the behavioral paradigm with a vertical slit formed by glass walls**. The upper part of the space between the vertical walls is covered so that the height of the slit is 20 mm.

The experiments were made on 13 female Wistar and 5 Sprague-Dawley rats (age 2–3 months, weight 300–400 g). They were housed in groups of six to eight in a cage (1.5 m wide, 0.5 m deep, and 0.5 m high) with grid walls and enriched with bedding, tubes, hammocks, and shelters. A test box (25 cm wide, 30 cm deep, 35 cm high; c.f. Figure 1) for behavioral experiments was directly attached at the end of the home cage.

### Behavioral test

The animals were trained in a behavioral paradigm testing the ability to remove a morsel of food placed 10–15 mm behind a vertical slit. The rats were first familiarized with a simplified test by allowing the entire group, for three nights (separated by 48 h), *ad libitum* retrieval of crushed morsels of their regular food pellets (Harlan Teklad) through a slit in the front wall of the test box. The morsels were contained in a vertical cylinder with a hole placed directly behind the slit. Thereafter, the animals were trained at daytime in a setup mounted in the test box only during experiments. It resembled that of Whishaw et al. (8), but with the difference that the slit was not positioned in the front wall of the test box but was located between glass walls (1 mm thick) protruding 55 mm from the front wall (c.f. Figure 1). The bottom wall was positioned 23 mm above the floor. The farther side wall (background) was painted black to improve the contrast of the paw in the video images. The height of the slit was 20 mm and the width 13 mm. The intention of this arrangement was to allow for comparison with a test in the cat, developed by Górska and Sybirska (13), with retrieval of food from a horizontal tube at shoulder level. The morsels consisted of cut pieces (about 2 mm × 3 mm) of Rotastak Milk Drops (Armitage Pet Care). During the experiments, the home cage was separated into two parts with a slideable hatch so that only animals kept in the part to which the test box was attached could enter it and perform the test. In the first experiment, the entire group of rats had access to the test box but this number was successively reduced in the next two to five experiments so that the animals were normally tested one at a time. Occasionally, two animals had access to the test box and were tested in the same experiment. The rats were moderately fasted during the preceding night (about 10 pellets of their regular food and 2 dl of glucose solution were left in the cage). The preoperative training period consisted of 10–20 sessions with a minimum inter-session interval of 2 days. Each experiment continued until the animal was satiated (20–100 trials).

**Figure 2:**
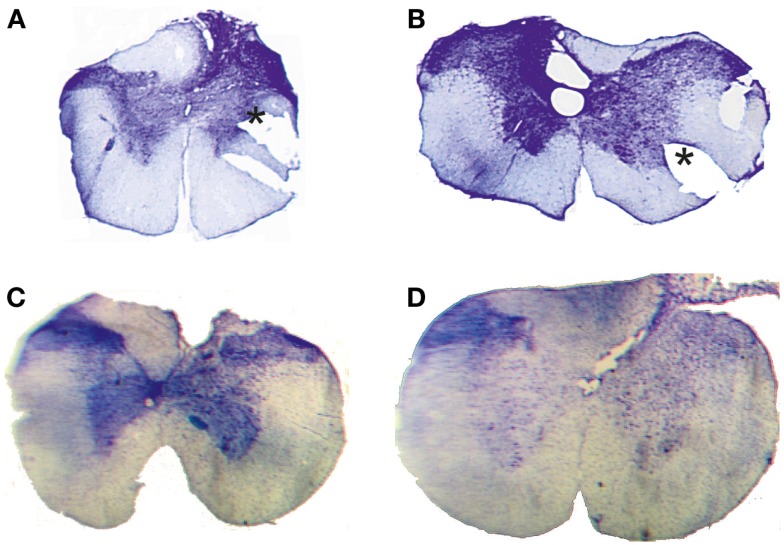
**Histological control**. Transverse extent of spinal selective lesions in four rats **(A–D)** giving complete interruption of the CST but with remaining fibers in unlesioned areas of the DC. The asterisks in **(A,B)** show the position of a notch cut, after fixation, in the lateral funicle on one side to identify that side of each section when mounting them.

Of the four animals, the behavior of which are described in this study, three (corresponding to lesions in Figures 2A,C,D) performed the movement with the left forelimb and the fourth animal (Figure 2B) with the right forelimb. In two of them (Figures 2A,B), the movements were recorded with a Motionscope PCI camera system (Redlake Imaging, San Diego, USA) with parallel sampling (two cameras, 250 Hz sampling rate, shutter 1/1250; sampling period 2 s) of views from above and from the lateral side. The entire experiments were also recorded on digital video cameras (40 Hz, Panasonic, Japan). In the two others (Figures 2C,D), the movements were recorded with digital video cameras (100 Hz, Sony, Japan).

### Movement analysis

The movements were evaluated qualitatively from the video images. In addition, the success rate, defined as the percentage of trials in which the morsel was grasped and brought to the mouth [c.f. (9)] was calculated from all trials performed in an experiment. This parameter was 90% at the end of the pre-operative training. Postoperatively, the success rate was measured on the first day each individual animal participated in the test and which corresponded to day 8, 16, 11, and 7, respectively in the animals the lesions of which are shown in Figures 2A–D. In the one animal, which performed the arpeggio movement and which was filmed with high-speed video, the movement was rated according to Whishaw et al. (14) using a three-point scale (2 = arpeggio present, 1 = arpeggio slightly abbreviated but recognizable, 0 = arpeggio absent). Since the number of animals with adequate lesion was limited (*n* = 4; c.f. results) and high-speed video was used only in two of them, the material was considered too small for statistical analysis.

### Surgery and animal care

Anesthesia was induced by isoflurane after which Dormicum (i.p.), Hypnorm (i.p.), Atropine (s.c.), Carprofen (s.c.), and Ringer’s Acetate solution (s.c.) were administered. The animal was positioned on a heating pad, intubated, and artificially ventilated. The anesthesia was maintained with isoflurane using a Univentor unit (Agnthos, Lidingö, Sweden). Rectal temperature, sPO2, ECG, and end-tidal CO2 was continuously monitored. The border between the C1 and C2 vertebras was exposed by a midline incision and retraction of the dorsal neck muscles. In some cases, a small laminectomy was made of either the caudal part of C1 or the rostral part of C2. The dura was opened transversally and the dorsal column partially transected with watch-makers forceps. The forceps were inserted into the dorsal column contralateral to the intended side of lesion. Starting from the dorsal root entry zone, an oblique lesion was made in the contralateral dorsal column along the pia layer overlying the dorsal horn. When reaching the ventral part of the dorsal column the lesion was enlarged to a bilateral transection of that area. The neck muscles were sutured with Vicryl (4–0) and the skin with Monocryl (4–0) sutures. Buprenorphine (s.c.) and Ringer’s acetate solution (s.c.) were given immediately postoperatively. Vital values were monitored until sPO2 could be maintained above 95% with spontaneous breathing in room air. The animal was then put on a heating pad in a separate part of the home cage. During the first postoperative week, the animals were kept at least two together in an area of the home cage with a height reduced to 15 cm to restrict them from climbing on the grid walls.

### Control of lesions

#### Electrophysiology

The completeness of the CST lesion was assessed in acute electrophysiological experiments. The preparation has previously been described in detail Alstermark et al. (11). In brief, the animals were anesthetized with a mixture of fentanyl and midazolam (2.8 ml/kg i.p.) and then by α-chloralose (60 mg/kg i.v.). Atropin (0.5 mg), dexamethasone (2 mg) was administered just after the induction of anesthesia. During recordings, the animals were immobilized with pancuronium bromide and artificially ventilated. The rostral part of the C1 and the C4-Th1 spinal segments were exposed by laminectomy and a posterior craniotomy was performed over the cerebellum to allow for insertion of stimulating electrodes in the pyramid. Corticofugal fibers were stimulated in the contralateral pyramid at 0.5 mm lateral to the midline, 2 mm rostral to the Obex level with a rostral angle of 30° using tungsten electrodes. Recording of the descending volley was made from the surface of the DC, cord dorsum potential (CDP), rostral and caudal to the lesion using a silver ball electrode. Using glass micro-electrodes, intracellular records were obtained from forelimb motoneurons (MNs) in C6–C8, with a minimal membrane potential of 40 mV. All signals were sampled using a Digidata 1200 recording system and analyzed off-line with Clampfit (Axon Instruments, Foster City, CA, USA).

#### Histology

At the end of the acute experiments the animals were sacrificed with pentobarbital (i.v.) and perfused with 3% formaldehyde solution after which the spinal cord was removed. The C1–C2 segments were freeze-sectioned (50 μm), stained with cresyl violet and the transverse extent of the lesion evaluated histologically.

## Results

### C1–C2 CST/DC lesions

Complete transection of the CST with minimal damage to more dorsally located fibers was achieved only in 4 animals out of 18. In the other cases, which were excluded, the CST lesion was incomplete or the entire dorsal column was transected. Figure 2 shows the histological extent of the four successful C1/C2 DC lesions. In all cases, the lesion covered the most ventral part of the DC bilaterally were the CST is located. In addition, the lesions interrupted, to different extents, also more dorsally located fibers in the DC. On the side ipsilateral to the performing limb (right side in Figure 2B, left side in Figures 2A,C,D), the largest dorsal extent was found in lesion Figure 2B. In the other animals, the lesions were more confined to the ventral part of the DC.

### Electrophysiology

**Figure 3:**
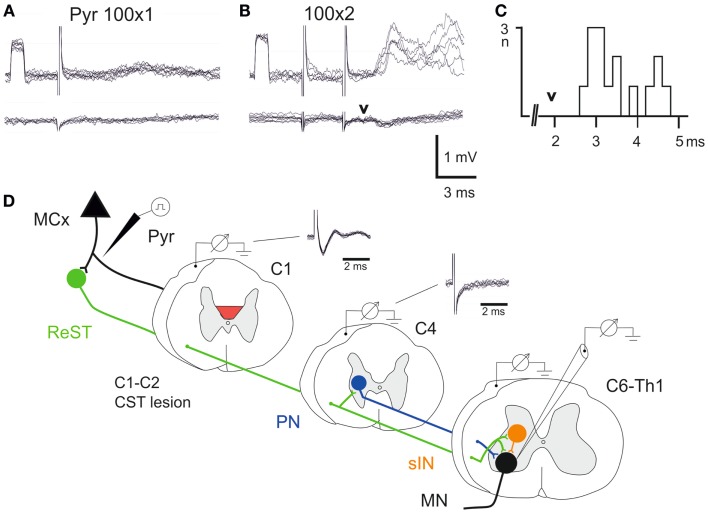
**Electrophysiological control**. **(A,B)**, upper traces are intracellular recordings from a MN antidromically identified by stimulation of the ulnar/median nerves. Lower traces were recorded from the cord dorsum in the same segment (C7) as the intracellular recordings. The contralateral pyramid was stimulated electrically with a single pulse at 100 μA in (**A)** and with two pulses in **(B,C)**, histogram of EPSP latencies measured from the second pyramidal stimulus to the onset. Arrow head indicates the arrival of the synaptic volley in C7. **(D)** Schematic circuit diagram of demonstrated cortico-motoneuronal pathways. ReST in green, PN pathway in blue and sINs in orange. Note that the ReST has both monosynaptic projection to MNs and disynaptic projection via PNs and sINs. The red area indicates the lesion of CST in C1–C2. Cord dorsum recordings from C1 and C4 evoked by a single stimulation in the contralateral pyramid at 100 μA.

In all animals, it was verified that the lesion had completely abolished the corticospinal volley recorded below the lesion. The experimental setup is outlined schematically in Figure 3D. The volley was recorded from the surface of the DC in C1 and C4. It can be seen that the lesion completely abolished the corticospinal volley in C4. The negative component (upward) was eliminated and only the stimulus artifact remained. In two animals (lesion in Figures 2C,D), intracellular recordings were made from forelimb MNs innervating wrist and digit extensor or flexor muscles. In Figures 3A,B is illustrated intracellular recordings from a flexor MN (upper traces).

A single electrical stimulus given in the contralateral pyramid (Pyr) evoked no effect, whereas a double pulse elicited excitatory postsynaptic potentials (EPSPs) as shown in Figure 3B. Latency measurements from the second electrical stimulus to the onset of the EPSPs are shown in Figure 3C. The latencies ranged between 2.5 and 4.6 ms (*n* = 15, 3.48 ± 0.17 ms). In the lower traces of the cord dorsum recordings, a small synaptic volley was observed (arrow head) after the second pyramidal stimulus. This synaptic volley had a latency of 1.9 ms from the stimulus as indicated by the arrow head in C. Thus, the shortest EPSP latencies shown in the histogram (2.5–3.0 ms) were in a monosynaptic range measured from the synaptic volley (below 1 ms), whereas longer latencies (3.0–4.0) were in a disynaptic range. The longest latencies (4.2–4.6) could be compatible with a trisynaptic range. The shortest latency EPSPs recorded after the C1–C2 CST lesion are compatible with transmission by reticulospinal neurons projecting directly to forelimb MNs (11), as shown in Figure 3D in green color. A disynaptic excitatory pathway could include both C3–C4 propriospinal neurons (PNs; blue) and sINs; orange that are activated by reticulospinal neurons (15). Thus, these findings are in agreement with those obtained after acute transection of the CST in the rat (11) and suggest that these pathways can operate also after a chronic CST lesion.

### Behavior

**Figure 4:**
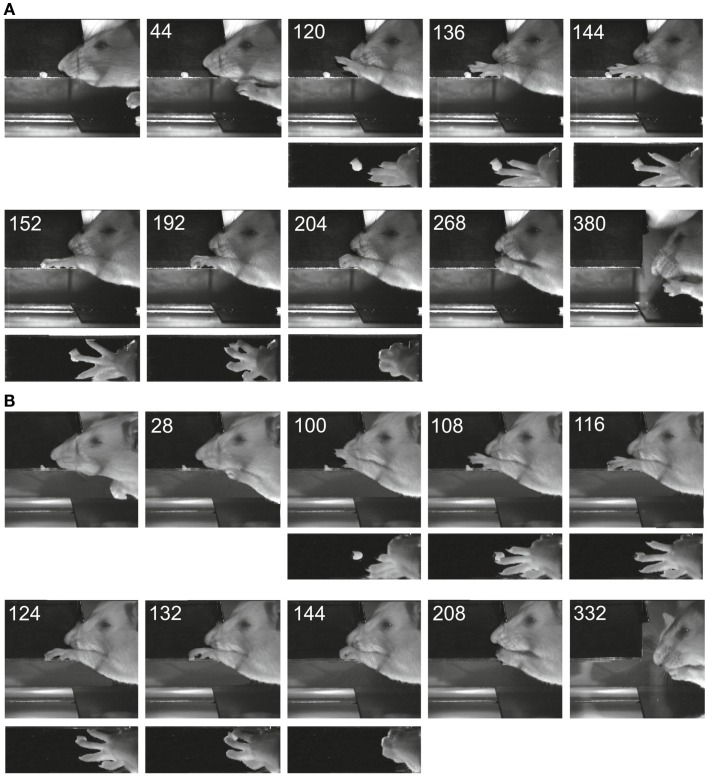
**Images of reaching and digit grasping movements obtained with high-speed video (250 Hz) preoperatively (A) and 8 days postoperatively (B) viewed from the lateral side and from above**. Times are given in milliseconds relative to the first image. The background has been digitally retouched.

Figure 4 shows two examples of reaching and grasping movements obtained preoperatively (Figure 4A) and on the 8th postoperative day (Figure 4B) in the same animal (lesion shown in Figure 2A). Video sequences from the same rat are illustrated in Movies S1 and S2 in Supplementary Material (pre- and post-operative). It is evident that, in both cases, the animal was able to reach for the morsel of food without signs of dysmetria, to perform a digit grasping movement resulting in successful retrieval and to bring the morsel to the mouth. Both pre- and post-operatively, digit grasping involved preparatory extension and abduction of the digits (frames 120 ms pre-operatively and 100 ms post-operatively) and an arpeggio movement (16) i.e., a combination of pronation and digit flexion, during which the digits were successively put down on the surface, starting with digit 5 (frames 136, 144, and 152 ms pre-operatively and frames 108, 116, and 124 ms post-operatively. In both movements, the morsel was first touched by digit 3 and then grasped by combined flexion and adduction of the digits. Then the paw was supinated before bringing the food to the mouth. The pre- and post-operative movements are markedly comparable and note the remaining supination, which was lacking following pyramidotomy (8). In the present study, supination was evident in the post-operative movements invariably and the paw was never put down pronated (flat) onto the floor after retrieval. The post-operative arpeggio movements in this rat resembled those observed pre-operatively and the mean rating on a three point scale (c.f. methods) was 1.3 (*n* = 22) pre-operatively and 1.4 (*n* = 12) in the first post-operative experiment. Similar findings were made in the other rats with the exception that one of them (corresponding to the lesion in Figure 2B) did not perform arpeggio movements in the test, neither pre- nor post-operatively. The success rate in each individual animal on the first post-operative day in which the animal participated in the test was 94% (Figure 2A, day 8), 96% (Figure 2B, day 16), 96% (Figure 2C, day 11), and 88% (Figure 2D, day 7).

## Discussion

The present results suggest that the control of reaching and grasping with the forelimb in the rat is not critically dependent on spinal circuits controlled by the CST. Following the CST lesion at C1–C2 levels, all the rats showed similar success rate as before the lesion in reaching and grasping the piece of food with the forepaw. Our results differ from previous findings suggesting a role of the CST in the control of the forelimb (8, 9). Earlier, much emphasis was given to a direct excitatory cortico-motoneuronal that was considered to provide a high degree of dexterity in the rat (10), but later it was proposed that also indirect cortico-motoneuronal CST pathways were important (9, 11). In fact, it was shown that there is no such direct pathway. Instead, the fastest excitatory pathway from the motor cortex is mediated disynaptically via a cortico-reticulospinal–motoneuronal pathway, whereas long latency excitation is mediated via sINs (11, 12). Our present electrophysiological control experiments confirm that disynaptic excitation could still be evoked in forelimb MNs after the chronic CST lesion. One explanation for the different results in the present study compared to those of Whishaw et al. (8) and Alaverdashvili and Whishaw (9), may be that in the latter two studies the pyramidal lesion and motor cortex lesion eliminated the cortical input to reticulospinal neurons as well as to the spinal cord.

Since our lesions did not interrupt rubral pathways, we do not exclude that they may also contribute to this forelimb motor control. It was shown that lesions of the nucleus ruber resulted in defective control of reaching and paw movements (14), especially the searching (arpeggio) component during pronation (17) and the importance of the rubrospinal tract in the control of grasping was demonstrated in the cat (4). Interestingly, Whishaw et al. (14) found that even following combined lesions of the pyramid and of nucleus ruber, the rats could still reach and grasp despite their deficits. Whishaw et al. (14) in fact emphasized that “*some components of skilled limb use are supported by descending neural pathways or spinal cord circuits other than the crossed rubrospinal or corticospinal projections*.” Our results suggest that one candidate could be the cortico-reticulospinal pathway.

From a phylogenetic perspective, it is interesting that there is a striking similarity in the kinematics of reaching and grasping in the rat and mouse (18). In the mouse, it was recently shown that reticulospinal neurons in the lower brain stem are important in the control of these movements (19). This finding is supported by electrophysiological experiments using intracellular recordings from adult mouse forelimb MNs that demonstrated a disynaptic cortico-reticulospinal excitatory pathway (20). In contrast to the rat, the CST evoked excitation in mouse forelimb MNs was much weaker and less frequent (20). These authors proposed that the CST may be less involved in the control of MNs, but may be more so in the control of segmental reflex systems in the mouse. The present results suggest that the same be true in the rat. It appears that there is a gradual expansion in the control of spinal circuits by the CST during phylogenesis, with a weak control in the mouse, stronger in the rat of sINs, even stronger in the cat with additional projection to C3–C4 PNs and strongest in primates with the additional direct CM projection (21). In contrast, the cortico-reticulospinal input still remains throughout these species although it is becoming weaker. Even so, the cortico-reticulospinal pathway to forelimb MNs was shown to be highly plastic and could be strengthened after partial spinal cord lesion involving both the CST and rubrospinal tract in the cat (22). Our finding is of interest from a phylogenetic perspective since it shows that similar skilled movements like reaching and grasping can be controlled by different motor pathways in different species of animals.

## Conflict of Interest Statement

The authors declare that the research was conducted in the absence of any commercial or financial relationships that could be construed as a potential conflict of interest.

## Supplementary Material

The Supplementary Material for this article can be found online at http://www.frontiersin.org/Journal/10.3389/fneur.2014.00103/abstract

Movie S1 **Examples of pre-operative movements in the rat illustrated in Figure 4**.

Movie S2 **Examples of post-operative movements in the rat illustrated in Figure 4**.
